# Silica nanoparticles assisted Ba_2_SiO_4_:Eu^2+^—a bluish-green emitting remote phosphor for white light application

**DOI:** 10.1007/s12200-025-00150-w

**Published:** 2025-04-09

**Authors:** Abinaya Mayavan, Aarthi Kannan, Sakthivel Gandhi

**Affiliations:** 1 Department of Chemistry, School of Chemical and Biotechnology, SASTRA Deemed University, Thanjavur, 613401 India; 2 Centre for Nanotechnology & Advanced Biomaterials, SASTRA Deemed University, Thanjavur, 613401 India

**Keywords:** Remote phosphor, Barium silicate phosphor, Bluish-green emission, Silica nanoparticle

## Abstract

Silica nanoparticles were used to develop a bluish-green emitting Ba_2_SiO_4_:Eu^2+^ phosphor, demonstrating their potential for white light applications. The phosphor showed a 48% enhancement of emission intensity compared to conventional silica-assisted phosphors. The use of silica nanoparticles as a precursor could lead to the creation of a more homogeneous distribution of cations and dopant ions. This uniform distribution could facilitate the proper infusion of dopants into the crystal host, resulting in improved emission. The phosphor exhibited high thermal stability, with 56% of its luminescence intensity maintained even at 190 °C compared to room temperature. To reduce thermal stress, a flexible remote phosphor has been developed successfully using optimized silica nanoparticles assisted Ba_2_SiO_4_:Eu^2+^ phosphor.

**Graphical Abstract:**

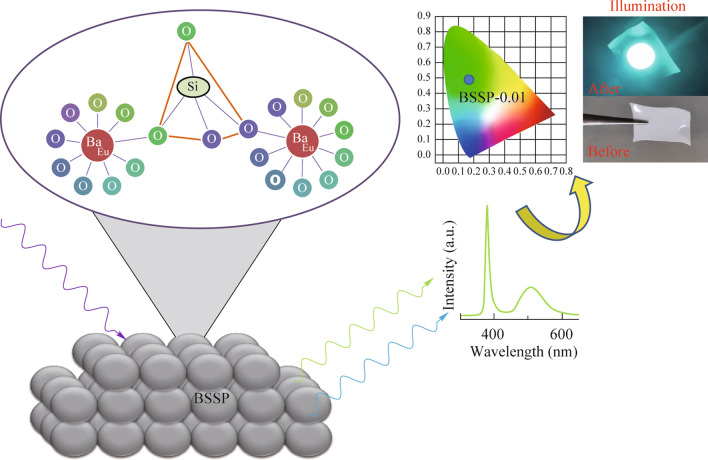

**Supplementary Information:**

The online version contains supplementary material available at 10.1007/s12200-025-00150-w.

## Introduction

White light-emitting diodes (LEDs) have garnered considerable attention for solid-state lighting because of their energy efficiency, small design, long lifespan, high efficiency, and environmental friendliness [1–4]. The commercialized white LEDs are usually based on InGaN LED chips with yellow emitting phosphor (Y_3_Al_5_O_12_:Ce^3+^) embedded in epoxy resin. The prolonged heat exposure may cause the resin to lose its original color, resulting in a yellowish tint and reduced performance in terms of brightness. This change in color and efficiency can negatively impact the overall performance and appearance of the LED [5, 6]. Recently, remote phosphor technology has become an alternative technology to reduce thermal stress. White LEDs have a remote phosphor arrangement in which the phosphor is at a considerable distance from the LED chip [7]. This arrangement may increase the phosphor efficiency by lowering the amount of light that is backscattered onto the LED chip [8]. The development of film using oxide phosphor with thermally stable and highly luminescence efficient are currently in high demand.

Especially, white LEDs utilizing europium-doped silicate-based phosphors and gained more attention from researchers because of their outstanding chemical and thermal stability, water resistance, good concentration quenching properties, cost-effectiveness, strong absorption in the UV range, excellent luminescent properties [9, 10]. Due to their comparable ionic radii, divalent europium is more stable in an alkaline earth silicate host and diffuses into the lattice sites more readily [11–17]. As a member of the silicate phosphor family, Barium orthosilicate (Ba_2_SiO_4_) has advantages such as higher physical and chemical stability, long serving life, lower harm to the health and environment, and easy fabrication [18]. So, Ba_2_SiO_4_ is a well-known phosphor host and has been widely studied. Different kinds of silica precursors have been used by various researchers in the development of barium silicate, such as conventional silica, fumed silica, tetraethyl ortho silicate, and sodium metasilicate.

Our earlier research employed the same approach and relied on the synthesis of silica nanoparticles, which were then used as a silicate source to develop Ca_2_SiO_4_:Eu^2+^ [19], CaSrSiO_4_:Eu^2+^ [20], and Ba_2_SiO_4_:Eu^3+^ [21] and showed increased luminescence efficiency compared to conventional silica assisted silicate phosphors.

We now extend our investigation to silica nanoparticles assisted Ba_2_SiO_4_ phosphor. The utilization of silica nanoparticles offers a unique pathway to tailor the optical properties of Ba_2_SiO_4_ phosphor. Silica nanoparticles, with their high surface area improves the luminescence intensity. In this work, a series of silica nanoparticles assisted Ba_2_SiO_4_ phosphors was prepared by varying the Eu^2+^ concentration, by adopting a dry phase solid-state reaction method, and compared their efficiency with conventional silica assisted Eu^2+^ doped Ba_2_SiO_4_ phosphor. In addition, to reduce thermal stress, remote phosphors have been developed using optimized silica nanoparticles assisted Eu^2+^ doped Ba_2_SiO_4_ phosphors and studied for their luminescence efficiency with respect to their weight ratio.

## Materials and methods

### Synthesis of silica nanoparticles

The synthesis of silica nanoparticles adopts Stober’s process [22]. Initially, the calculated amounts of ethyl alcohol and distilled water were taken in a beaker. The silica precursor, tetra ethoxy silane (TEOS) (*Sigma Aldrich,* 99.9%), were added slowly to the solution containing the ethyl alcohol-H_2_O mixture. The solution was allowed to be stirred to attain homogeneity. Ammonium hydroxide (NH_4_OH) (*Sigma Aldrich,* 25%) was added to the above solution which leads to precipitation. The precipitated white silica nanoparticle was collected by washing with ethyl alcohol after centrifugation. Finally, it was dried at room temperature and named as SNPs.

### Synthesis of Ba_2_SiO_4_:Eu^2+^ phosphor

All Ba_2_SiO_4_:Eu^2+^ with different doping concentrations (0.007, 0.008, 0.009, 0.01, 0.02, and 0.03 mol) were prepared using a high temperature solid state synthesis method. The reactant materials contain BaCO_3_, Eu_2_O_3_ with 99.99% purity (*Sigma Aldrich*) and SiO_2_ nanoparticles. All the reactants were placed in agate and grounded for 1 h to get a homogeneous mixture, then the mixture was transferred into the crucible. The crucible was loaded into a box furnace and calcined at 1000 °C for 4 h to yield Ba_2_SiO_4_:Eu^3+^. The as-prepared Ba_2_SiO_4_:Eu^3+^ was again grounded and transferred into the alumina boat, which was sintered at 1200 °C for 4 h under a reducing atmosphere (10% H_2_/90% N_2_) to yield Ba_2_SiO_4_:Eu^2+^. The obtained samples were named as BSSP-0.007, BSSP-0.008, BSSP-0.009, BSSP-0.01, BSSP-0.02, and BSSP-0.03. Similarly, the conventional silica-assisted Ba_2_SiO_4_:Eu^2+^ phosphors were also prepared by using conventional silica with mesh size of 100 (*Sigma Aldrich,* 99.9%) as the silica precursor and the obtained samples were named as BSCP-0.007, BSCP-0.008, BSCP-0.009, BSCP-0.01, BSCP-0.02, and BSCP-0.03.

### Remote phosphor fabrication

The stoichiometric amount of silicon resin was mixed and kept in a vacuum desiccator to remove air bubbles. Then, the phosphor with different weights (mg) (30, 40, 50, 60, and 70) were taken and mixed with the silicon binder. The phosphor-silicon resin mixture was made into a thin film via drop casting.

### Characterization

The XRD data was collected from 20° to 80° with a scanning speed of 1 s and a step size of 0.02°. The morphologies of prepared phosphors were investigated using the field emission scanning electron microscopy (FE-SEM) instrument, Merlin compact-30 kVA (Germany). The photoluminescence excitation (PLE) and emission spectra (PL) were obtained using a solid-state fluorescence spectrophotometer equipped with a 150 W Xe lamp source (FP- 8300, Jasco, Japan). UV–visible (UV–vis) spectra was analyzed to compute optical band gap energy using UV–vis 750 spectrophotometer. Temperature-dependent photoluminescence was also recorded using a laboratory-made temperature controller and ocean optics spectroscopy (Ocean FX, Ocean Optics, Netherland). The CIE parameters (*x*, *y*) were calculated from the wavelength range of 380–750 nm and associated with the 1931 CIE standard. X-ray photoelectron spectroscopy (XPS) was used for the elemental analysis (K alpha surface analysis, Thermo Scientific).

## Results and discussion

### Crystal study

**Fig. 1:**
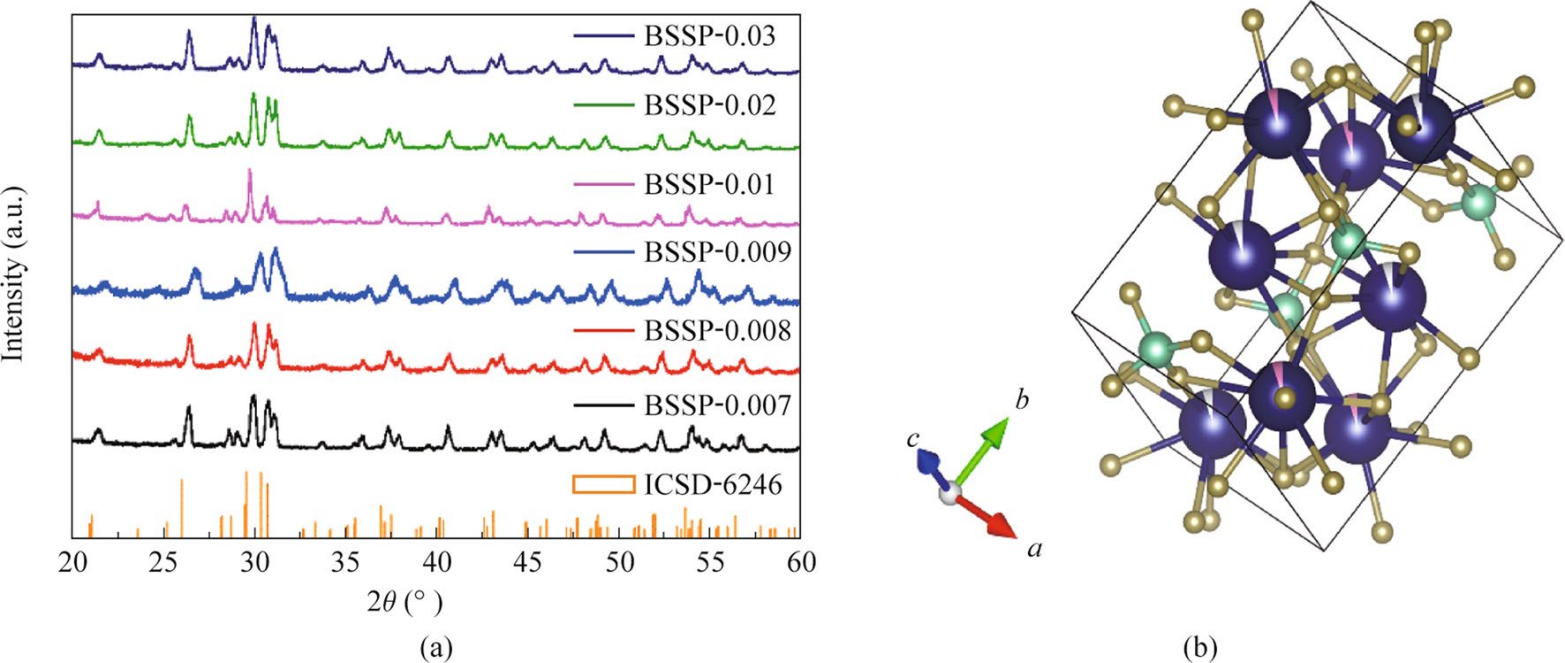
**a** X-ray diffraction patterns of BSSP-0.007, 0.008, 0.009, 0.01, 0.02, and 0.03 phosphors. **b** Schematic crystal structure of BSSP phosphor

The purity of the crystal phase of the developed phosphors was determined using X-ray powder diffractometry with Cu-K*α* radiation at 40 kV and 30 mA. The collected XRD patterns in the 20° − 80° range showed several diffraction patterns associated with a single-phase crystalline structure of Ba_2_SiO_4_ with an orthorhombic structure and space group Pmcn (as shown in Fig. 1). The recorded diffraction pattern of Ba_2_SiO_4_:Eu^2+^ phosphor, prepared using a solid-state reaction method, matched well with the reference pattern (ICSD-6246) and the presence of diffraction peaks corresponding to hetero-phase was not detected in the XRD patterns, even after doping at higher concentrations of Eu^2+^. These results suggest that the solid-state reaction method resulted in the production of crystalline Ba_2_SiO_4_:Eu^2+^ phosphor. Notably, the diffraction peaks of all the prepared phosphors shifted to larger angles due to the substitution of larger Ba^2+^ ions with smaller Eu^2+^ ions. It is worth mentioning that Ba has two sites with 9 and 10 coordination numbers. As a result, when Eu^2+^ is doped into Ba_2_SiO_4_, it has two possible sites to occupy within the crystal structure. The XRD pattern of BSCP phosphors with different concentrations of dopant are given in Fig. S1, suggesting the formation of Ba_2_SiO_4_ crystals. Like BSSP, BSCP also shows similar shifts in the XRD pattern due to doping of Eu^2+^ in place of Ba^2+^.

The schematic crystal structure of BSSP phosphor was obtained by the standard card as shown in Fig. 1b. In the figure, royal blue-golden color represents Ba–O, cyan-golden color represents Si–O, silver and pink color represent Eu atom. It is suggested that Eu has two occupying sites (Ba1 and Ba2) in the crystal lattice. The average crystallite size was calculated using Scherrer formula and found to be 36.4 nm. This nanoscale crystalline size suggests that the BSSP phosphor possesses a high surface area leads to enhancement of luminescence efficiency.

### Photoluminescence excitation and emission

**Fig. 2:**
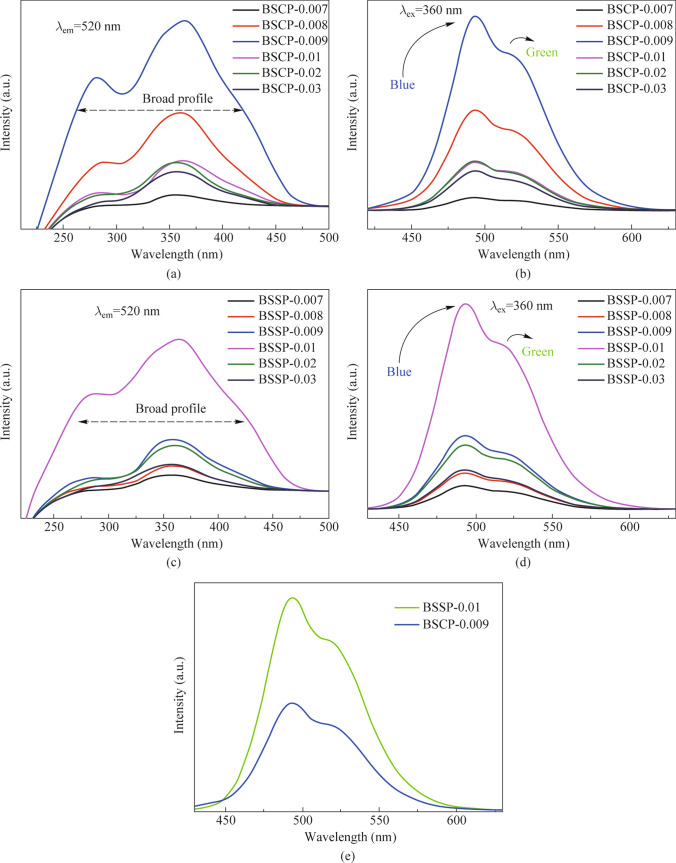
**a** and **c** Photoluminescence excitation spectra of BSSP & BSCP (−0.007, 0.008, 0.009, 0.01, 0.02, and 0.03). **b** and **d** Photoluminescence emission spectra of BSSP & BSCP (−0.007, 0.00, 0.009, 0.01, 0.02, and 0.03). **e** Comparative study of photoluminescence emission spectra of BSSP-0.01 and BSCP-0.009

Figure 2a and c show the recorded excitation spectra of BSSP-0.007, BSSP-0.008, BSSP-0.009, BSSP-0.01, BSSP-0.02, and BSSP-0.03 & BSCP-0.007, BSCP-0.008, BSCP-0.009, BSCP-0.01, BSCP-0.02, and BSCP-0.03 at an emission wavelength of 520 nm. A broadband within the range of 250 − 450 nm was observed, which was attributed to the 4f^7^ − 4f^6^5d^1^ transition [23]. The peaks of BSSP and BSCP were similar with no noticeable red or blue shifts. All the prepared phosphors have similar spectral features, except for changes in excitation intensities as the concentration of Eu^2+^ increases. These results suggest that the prepared phosphors exhibit strong absorption of UV and blue light, making them potentially suitable for use in UV and blue LEDs. Although there was no significant difference observed in the absorption range between BSSP and BSCP, there was a slight difference in the optimal concentration of dopant. This suggests that the incorporation of Eu^2+^ within the host is influenced by the precursor used.

The photoluminescence emission spectra of the prepared phosphors (BSSP-0.007, 0.008, 0.009, 0.01, 0.02, and 0.03 & BSCP-0.007, 0.008, 0.009, 0.01, 0.02, and 0.03) were recorded at an excitation wavelength of 360 nm and depicted in Fig. 2b and d. A broad emission was observed in the range of 450 − 650 nm, which corresponds to the 4f^6^5d^1^ − 4f^7^ transition. The emission spectra displayed two bands peaking at the wavelengths of 490 and 525 nm, which may be due to the presence of dopant in two different environments. The emission intensity gradually increased with an increase in Eu^2+^ doping from 0.007 to 0.01 mol. The maximum intensity was achieved at a concentration 0.01 mol of Eu^2+^. However, further increases in the Eu^2+^ concentration led to a decrease in the emission intensity, which may be attributed to concentration quenching, as shown in Fig. 2c and d. The optimized doping concentration was found to be 0.01 mol in BSSP and 0.009 mol in BSCP. Figure 2e shows the comparative emission studies of BSCP-0.009 and BSSP-0.01, which reveal that the 48% enhancement of emission intensity of BSSP-0.01 compared to BSCP-0.009, and the reason may be due to the use of nanoparticles as precursor. The utilization of silica nanoparticles as a precursor could lead to the creation of a more homogeneous distribution of cations and dopant ions. This uniform distribution could facilitate the proper infusion of dopants into the crystal host, resulting in improved emission.

Quenching centers generally cause defects in materials, which results in luminescence and non-radiative recombination. Since the recombination of electron–hole pairs due to surface defects is the most significant mechanism for non-radiative relaxation in particles, the difference in emission intensity should therefore mainly be related to the defects which originate from surface states of the particles [24]. Thus, the number of electron–hole pair via radiate recombination of BSSP-0.01 is more than that in BSCP-0.009 phosphor. As the results, PL intensity of BSSP is higher than that of BSCP phosphor. The use of silica nanoparticles improved the radiative recombination in the BSSP phosphor. The improved radiative recombination may be attributed to the equal distribution of dopant in the host crystal. As nanosized silica was used as a precursor, the high surface area present in the silica allowed the cations and dopants to be evenly distributed through physisorption. Field emission scanning electron microscopy (FE-SEM) was used to study the surface morphology of both silica nanoparticles and the conventional silica. In Fig. S2a, the FE-SEM image shows the silica nanoparticles, which have a size of approximately 300 nm and were used as a silicate precursor for preparing BSSP phosphor. Figure S2b displays the FE-SEM image of conventional silica with bigger particle size and irregular morphology, which were used as a silicate source for preparing BSCP phosphor. Figure S3a and b present the FE-SEM images of BSSP-0.01 and BSCP-0.009, with both phosphors exhibiting a rod-like morphology that varies in size. BSSP-0.01 demonstrated greater intensity than BSCP-0.009, despite having the same morphology.

The broad emission band upon the excitation wavelength of 460 nm have deconvoluted into two Gaussian spectral profiles with two peaks at 487 and 514 nm, suggesting that Eu^2+^ ions substitute two different Ba sites in BSSP phosphor (Fig. S4).

There is larger ionic radii difference between Si^4+^ (0.4 Å) and Eu^2+^ (1.30 Å). Therefore, Eu^2+^ possibly prefers the Ba1 and Ba2 sites in the crystal lattice. In the prepared phosphors, Eu substituted by Ba was surrounded by two oxygen environments, i.e., 9 and 10, which give rise to two emission centers, resulting blue and green emission. The Eu(II)-O has a shorter bond length than that of Eu(I)-O, so the effect of nephelauxetic and ligand field splitting is greater for Eu(II)-O than Eu(I)-O. Higher covalency results in higher ligand field splitting. Therefore, the green emission corresponds to the Ba site surrounded by 9 oxygen environments. A concentration quenching study was undertaken for optimized BSSP-0.01.

### Concentration quenching study

**Fig. 3:**
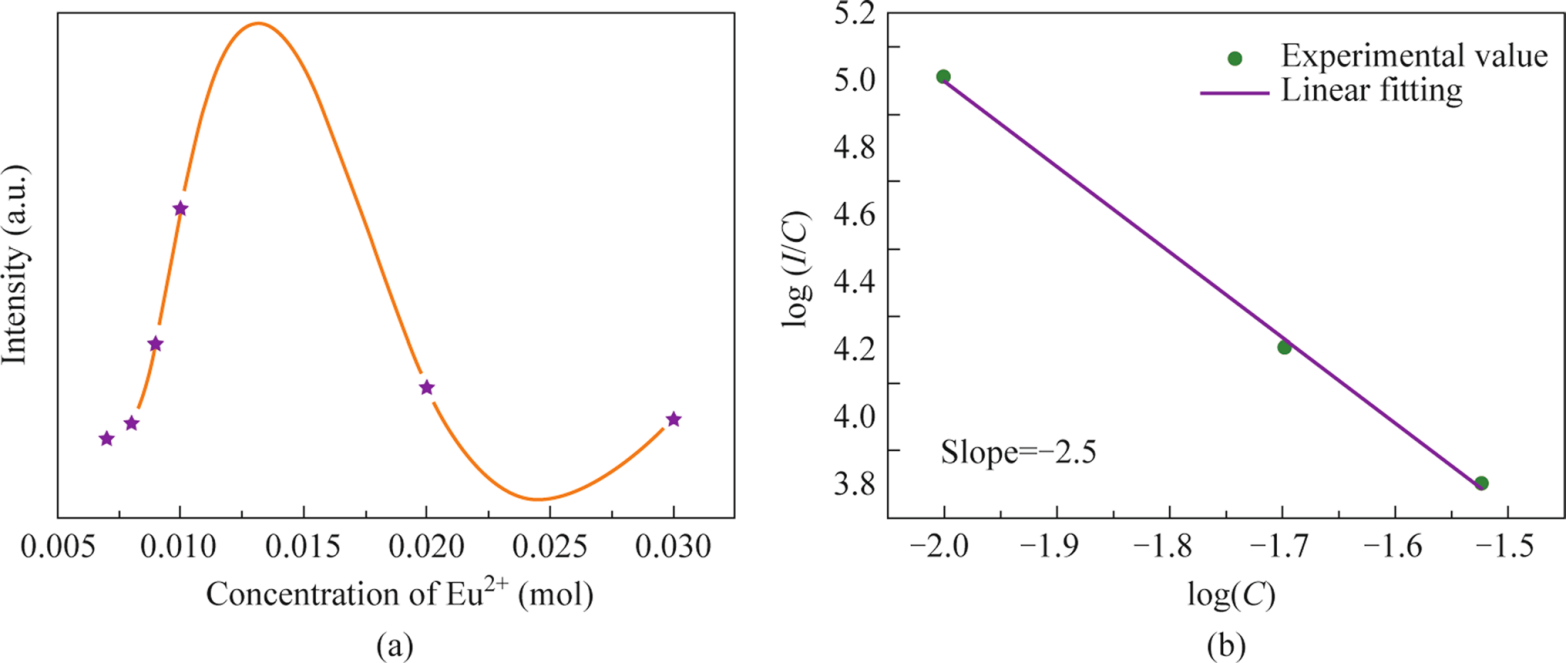
**a** Plot of the concentration of Eu^2+^ *vs* intensity. **b** Energy transfer mechanism plot of log(*C*) *vs* log(*I*/*C*)

To further investigate the concentration quenching mechanism, the critical distance (*R*_c_) between the activator and the quenching site was calculated. The oxide phosphor usually undergoes non-radiational energy transfer via exchange interaction or multipole-multipole interaction. If the distance between the activator was greater than 5 Å, energy transfer might occur due to multiple-multipole interaction [25]. The *R*_c_ of BSSP-0.01 phosphor was calculated by the equation given below [26, 27]

$${R}_{\text{c}}\sim 2(\frac{3v}{4\uppi {X}_{\text{c}}N}{)}^\frac{1}{3},$$

where $v$ denotes the unit cell volume, *X*_c_ stands for the critical concentration of Eu^2+^ ions where quenching occurs, and *N* indicates the number of Eu^2+^ ions that can occupy per unit cell. Here $v$, *N*, and *X*_c_ were 44.2 nm^3^, 4, and 0.01 mol, respectively. The calculated value of *R*_c_ for BSSP-0.01 was 28 Å, thereby revealing that the probability of concentration quenching in the BSSP phosphor via the multipole interaction mechanism was very high. To define the energy migration mechanism, the emission intensity per Eu^2+^ ions followed the Van Uiter equation.

$$\text{log}\left(\frac{I}{C}\right)=x-\frac{\theta }{3}*\text{log}\left(C\right),$$

where *I* represent the intensity of BSSP emission, *C* was the concentration of Eu^2+^. “*θ* = 3 represents the energy transfer that occurs amongst the neighbor ion, and *θ* = 6, 8, and 10 represent the energy transfer that occurs among the dipole–dipole (d-d) interaction, dipole-quadrupole (d-q) interaction and quadrupole–quadrupole (q-q) interaction.” Figure 3b depicts the linear relationship between log(*C*) *vs* log(*I*/*C*), the slope (− *θ*/3) was found to be − 2.5 and so the value of *θ* was 7.5, which is close to 8. The *θ* value demonstrated that the interaction type in BSSP phosphor was dipole– quadrupole ionic energy transfer.

### Temperature-dependent photoluminescence study

**Fig. 4:**
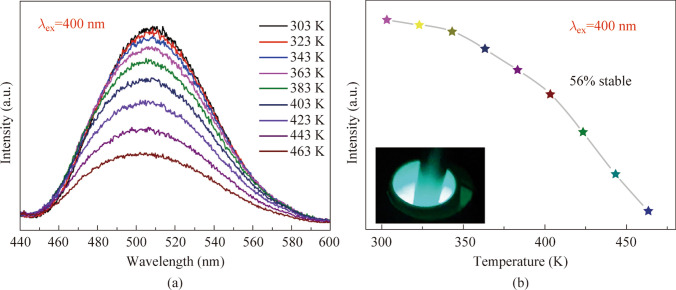
**a** Temperature-dependent photoluminescence spectra of BSSP-0.01 phosphor in the temperature range of 303–463 K. **b** Relationship between temperature and PL intensity, inset: Emission color of the phosphor under 400 nm excitation

Figure 4a illustrates the temperature-dependent photoluminescence study conducted on BSSP-0.01 phosphor to investigate its luminescent heat resistance. The emission intensity decreases with an increase in temperature (303 − 463 K), indicating temperature quenching. This mechanism involves the absorption of energy, leading to an electronic transition from the valence band to the conduction band. An energy level, known as a “trap”, exists between the valence and conduction bands. At higher temperatures, the trap level is thermally activated, resulting in a decrease in emission intensity. Figure 4b represents the graph plotted against luminescence intensity and temperature of the optimized phosphor. The thermal stability of the optimized phosphor was calculated to be 56% at 463 K, indicating its high resistance to thermal degradation compared to room temperature.

### CIE chromaticity

The chromaticity diagram and CIE coordinates were critical parameters for determining the phosphor’s emission color. The improved phosphor’s color point is highlighted on the CIE chromaticity diagram in Fig. S4. Under the excitation wavelength of 360 nm, the color coordinates (*x*, *y*) were found to be (0.15, 0.47). The BSSP doped with Eu 1 mol% emitted a bluish-green emission, as indicated by the CIE diagram (shown in Fig. S5).

### Band gap analysis

**Fig. 5:**
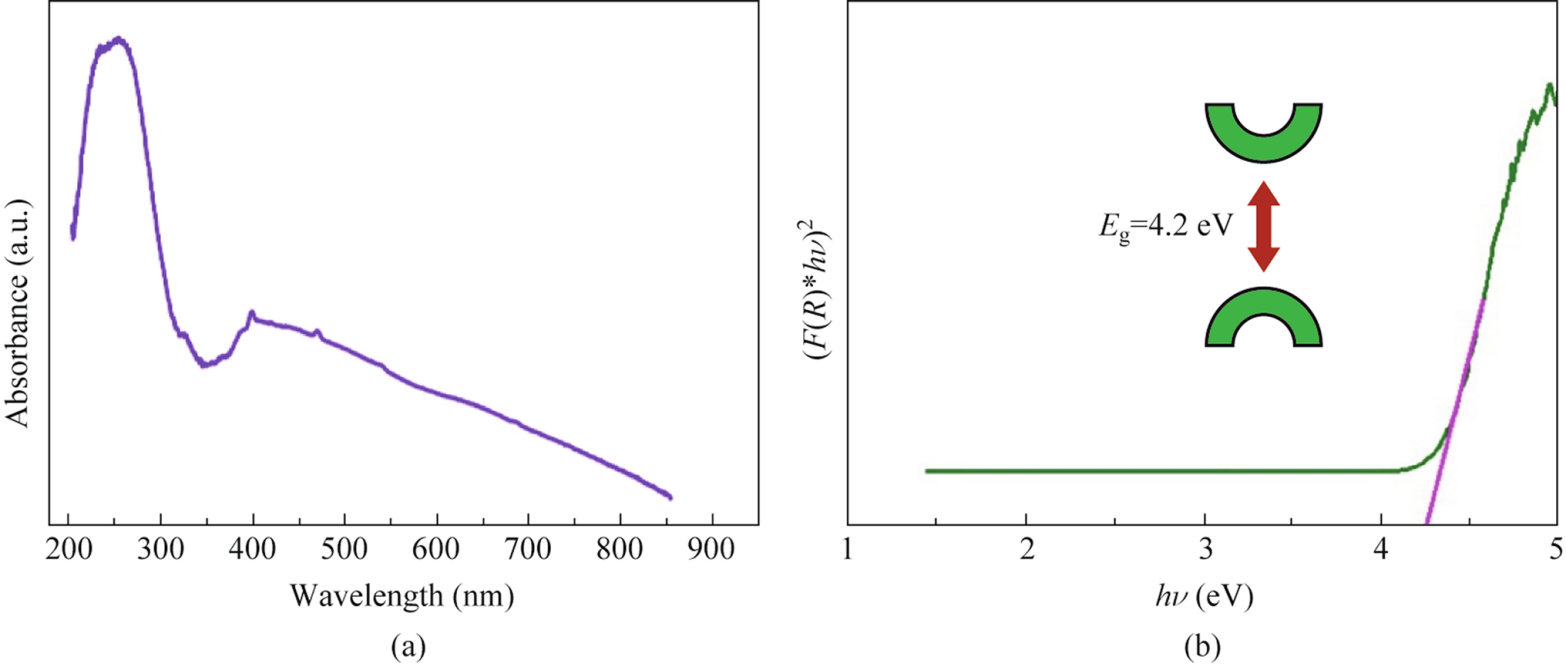
**a** UV–vis spectra. **b** Band gap plot of BSSP-0.01 phosphor

UV–vis spectra were employed to determine the band gap of phosphor materials. The UV–vis spectra of BSSP-0.01 phosphor are depicted in Fig. 5a, exhibiting a broad absorption peak from 200 to 350 nm, attributed to the electronic transition from 4f to 5d of Eu^2+^. The energy band gap was assessed using the Wood and Tauc methods. The Taucs plot in Fig. 5b illustrates an optical band gap of 4.2 eV. Notably, the band gap value of BSSP-0.01 is lower than that reported value in a previous study [25]. As the band gap decreases, more electrons from the valance band enter the conduction gap after being irradiated with incident light, resulting in increased emission intensity.

### Elemental analysis

**Fig. 6:**
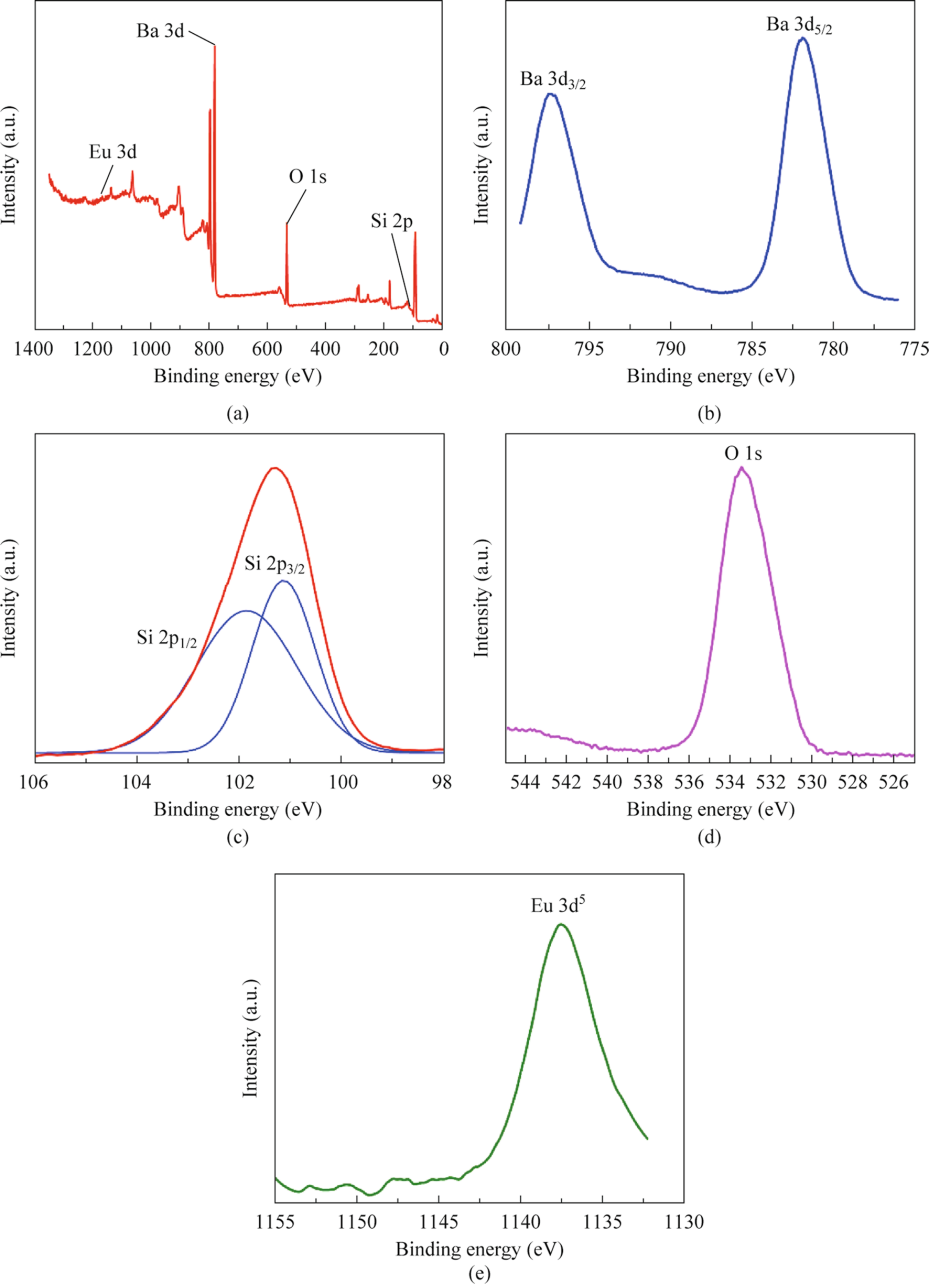
XPS spectra of BSSP-0.01. **a** Survey spectra. **b** Ba 2p spectra exhibiting two peaks such as Ba 2p_1/2_ and Ba 2p_3/2_. **c** Si 2s spectra showing two peaks Si 2p_3/2_ and Si 2p_1/2_. **d** O 1s spectra showing binding energies at 533 eV. **e** Eu 3d^5^ spectra display binding energy at 1137 eV

The presence of silicon, oxygen, barium, and europium elements were confirmed by the XPS survey spectra of BSSP-0.01 (Fig. 6a). The Ba^2+^ spectra shown in Fig. 6b were distinguished by two peaks at 781 and 797 eV. Due to orbital splitting, Ba ions produce two peaks: Ba 2p_3/2_ and Ba 2p_5/2_. Figure 6c depicts the binding energy of the O 1s electron, which was peaked at 533 eV. The Si spectra in Fig. 6d were distinguished by two peaks. The binding energies of two peaks, which correspond to Si 2p_3/2_ and Si 2p_1/2_, are 101.1 and 101.9 eV, respectively. These peaks could be attributed to Si–O or Si–O-Si bonding [28]. Figure 6e depicts the Eu 3d spectrum at 1137 eV, corresponds to Eu in the +3-oxidation state. Because Eu^2+^ was thermodynamically unstable, when the phosphor is subjected to X-rays, oxidation occurs, resulting in the formation of Eu^3+^ [19].

### Remote phosphor

#### Concentration *vs* luminescence study

**Fig. 7:**
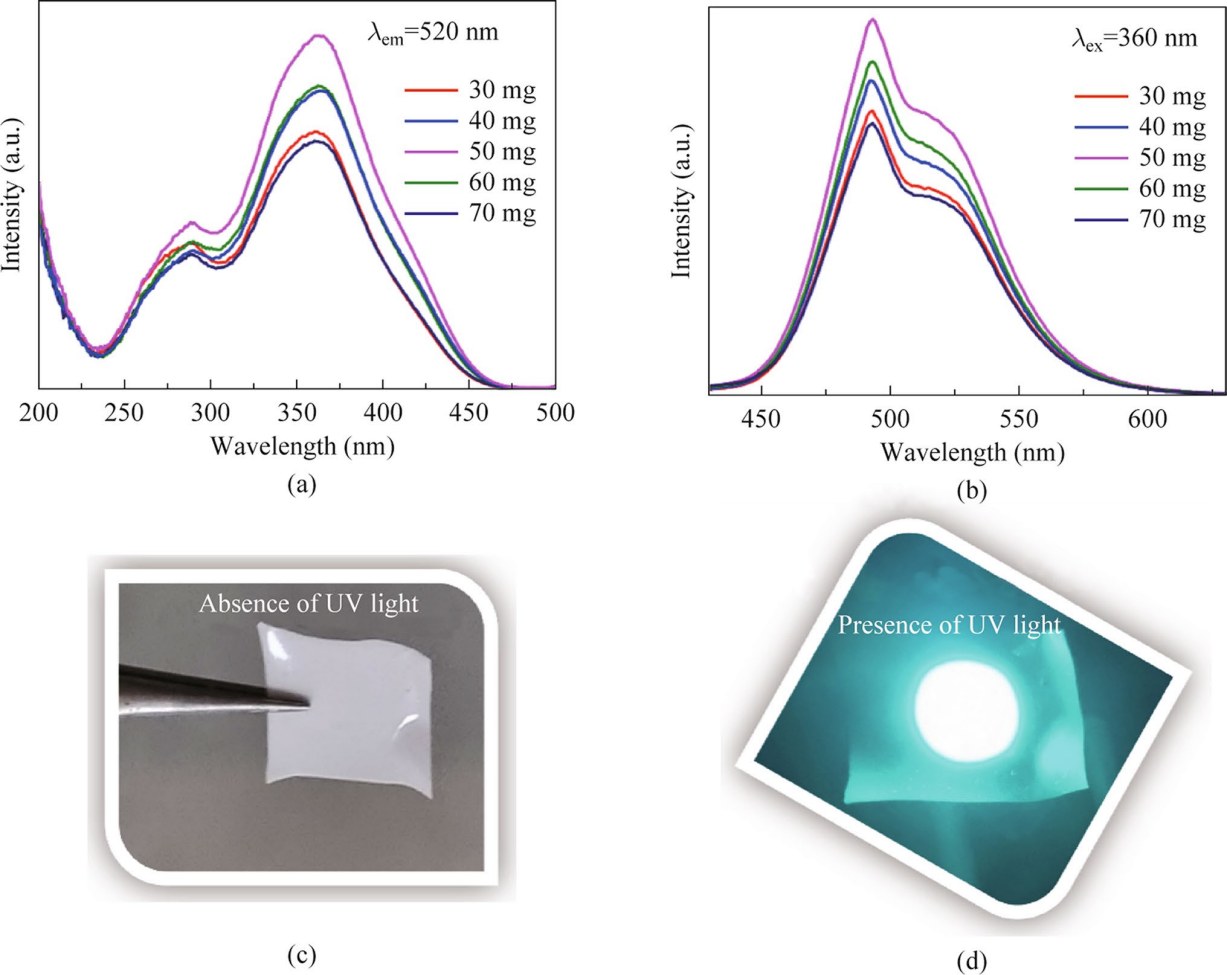
**a** and **b** Study between the weight of the phosphor and the change in luminescence intensity. **c** and **d** Illumination effect of the prepared BSSP film in the presence and absence of UV light

The BSSP-0.01 phosphor was used to develop the remote-type phosphor (shown in Fig. 7), which was then tuned for its performance and emission. The optimum condition for the ratio of phosphor to silicon resins was identified after extensive study. As shown in Fig. 7, the weight of the phosphor (30, 40, 50, 60, and 70 mg) was adjusted along with the same ratio of silicon binder (60 mg), and their luminescence efficiency was examined. The intensity of the emission increased when the concentration was changed from 30 to 50 mg; however, beyond that point, it was discovered that the intensity decreased. This may be due to the scattering effect of the phosphor particle. The optimized weight of the phosphor was found to be 50 mg. The luminescence intensity was significantly influenced by the phosphor concentration (weight). The light extraction efficiency decreased as the concentration of the phosphor increased. In Table S1, the CIE for the various remote-type phosphors were provided. According to the results, the optimized remote-type phosphor was discovered to have CIE(*x*, *y*) coordinates of (0.15, 0.47) whereas the phosphor ratio’s alteration had no significant impact on CIE. This proves the color stability of phosphor at different concentrations.

#### Voltage-dependent photoluminescence

**Fig. 8:**
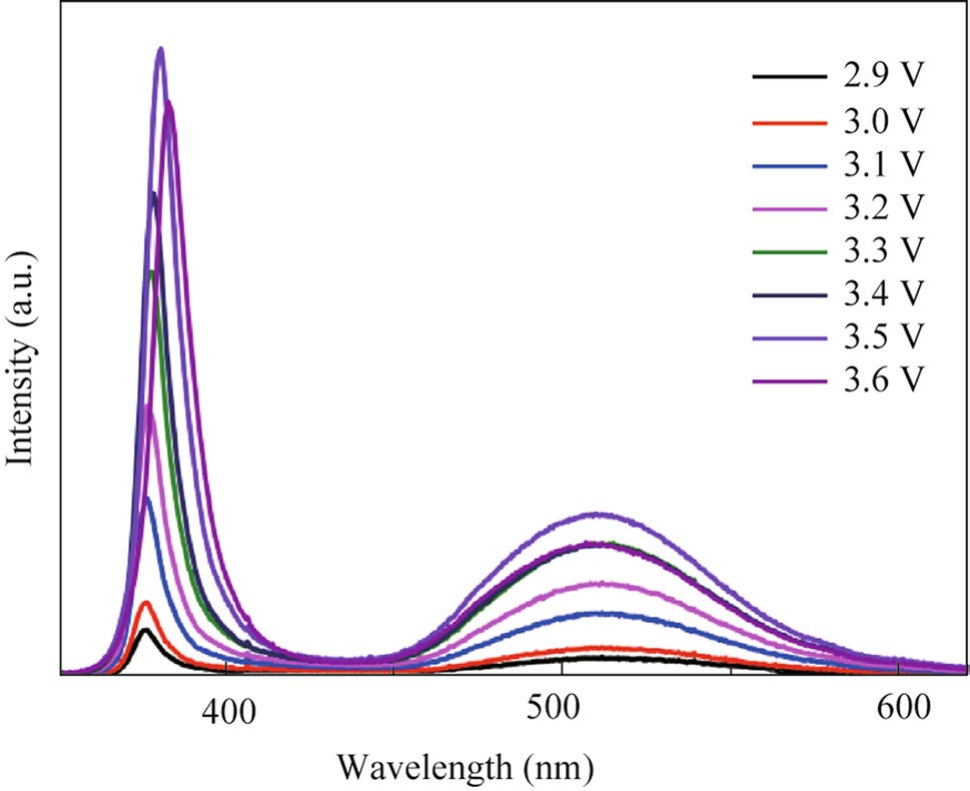
Voltage-dependent photoluminescence study of an optimized remote phosphor (BSSP film-50 mg)

Figure 8 depicts the white LED’s voltage-dependent color purity of BSSP film-50 mg. By changing the voltage from 2.9 to 3.7 V, the change in emission intensity of remote-type phosphor 50 mg was observed. When the applied voltage increases from 2.9 to 3.7 V, the emission spectra reveal a progressive increase in emission intensity. The emission curve’s shape did not significantly alter. When the voltage was raised from 2.9 to 3.5 V, an increase in intensity was observed; however, when the voltage was increased further, the intensity of the emission began to diminish (Fig. 8). The shape of the peak remains same even if the emission intensity decreases with increasing voltage over 3.5 V. This demonstrates the color stability at a range of applied voltages. The CIE coordinates were calculated and given in Table S2.

#### Real time application

**Fig. 9:**
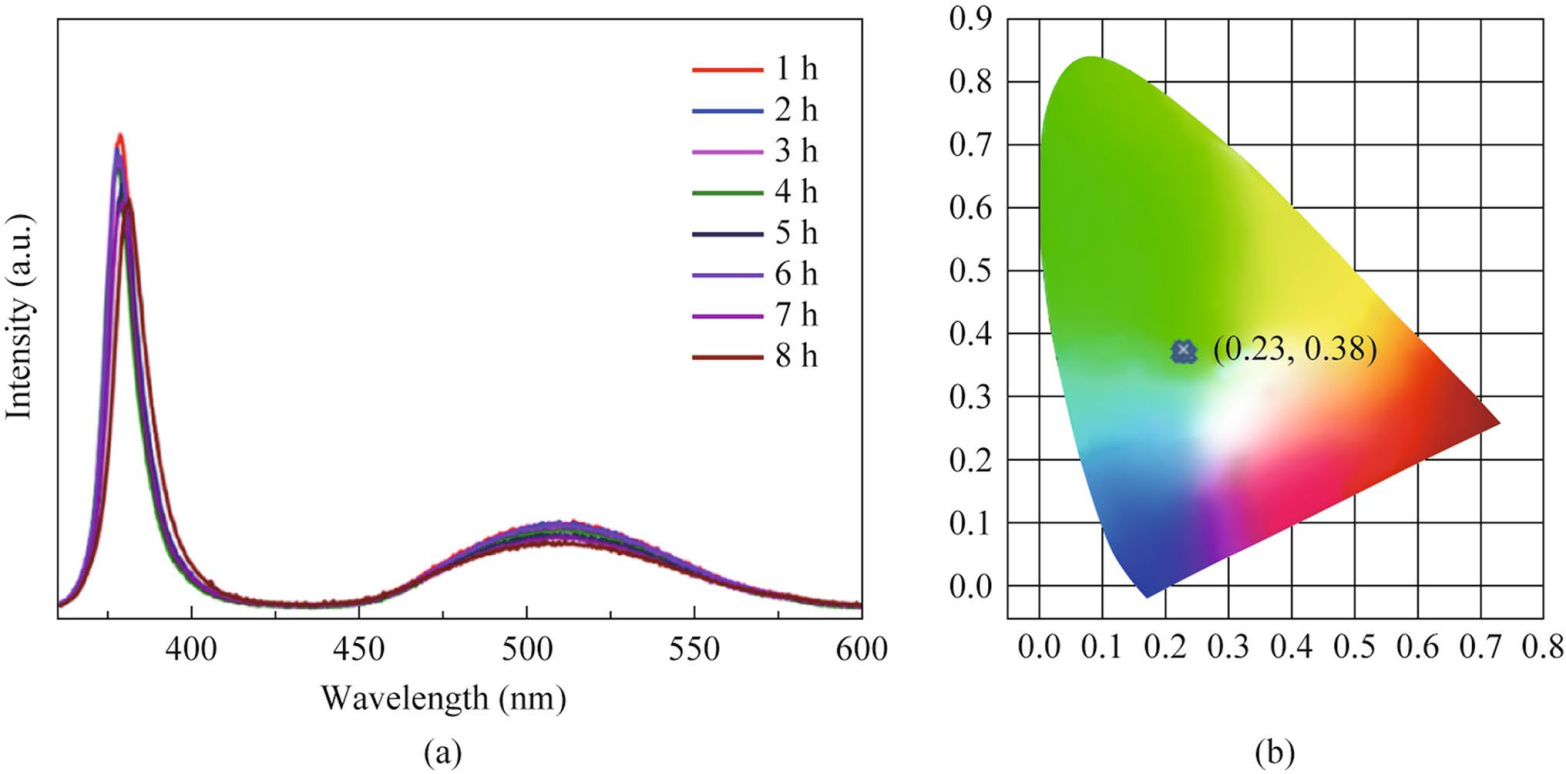
**a** Photoluminescence spectra. **b** CIE diagram of the optimized BSSP film-50 mg driven by 380 nm LED over a time

The optimized remote-type phosphor (BSSP film-50 mg) was placed on 380 nm UV LED prototype devices and observed their luminescence efficiency for 8 h (shown in Fig. 9). There was not much change in the intensity and emission shift were observed with respect to change in time. This implies that, the developed remote-type phosphor is highly stable. The calculated CIE coordinates are constant over the time as shown in Table S3.

## Conclusion

In summary, bluish-green emitting Ba_2_SiO_4_:Eu^2+^ phosphor was developed using silica nanoparticles as a silicate precursor for the first time. The use of silica nanoparticles as a silicate precursor has improved the efficiency of phosphor luminescence compared to the conventional silica particles. The prepared phosphor showed bluish-green emission with 56% thermal stability even at 190°C, compared to RT. To reduce thermal stress, a flexible remote phosphor has been developed successfully using optimized silica nanoparticles assisted Ba_2_SiO_4_:Eu^2+^ phosphor. The prepared remote phosphor showed good color stability at a range of applied voltage (2.9 − 3.7 V). The CIE coordinates were calculated and confirmed to be consistent over the time. This developed phosphor might be a promising choice for producing warm white light.

## Supplementary Information

Below is the link to the electronic supplementary material.

Supplementary material 1

## Data Availability

Not applicable.
