# Supplementary material for: Silica nanoparticles assisted Ba2SiO4:Eu2+—a bluish-green emitting remote phosphor for white light application
Source: Front Optoelectron. 2025 Apr 9;18(1):8. doi: 10.1007/s12200-025-00150-w (PMC11981998; doi:10.1007/s12200-025-00150-w)
Supplement: Supplementary file 1 — Supplementary material 1 [file 12200_2025_150_MOESM1_ESM.docx]

***Supporting Information***

**Silica nanoparticles assisted** **Ba_2_sio_4_: Eu^2+^ - a bluish-green emitting remote phosphor for white light application**

Abinaya Mayavan^1^, Aarthi Kannan^1^, Sakthivel Gandhi^1,2*^

^1^Department of Chemistry, School of Chemical and Biotechnology, SASTRA Deemed University, Thanjavur, India

^2^Centre for Nanotechnology & Advanced Biomaterials, SASTRA Deemed University, Thanjavur, India


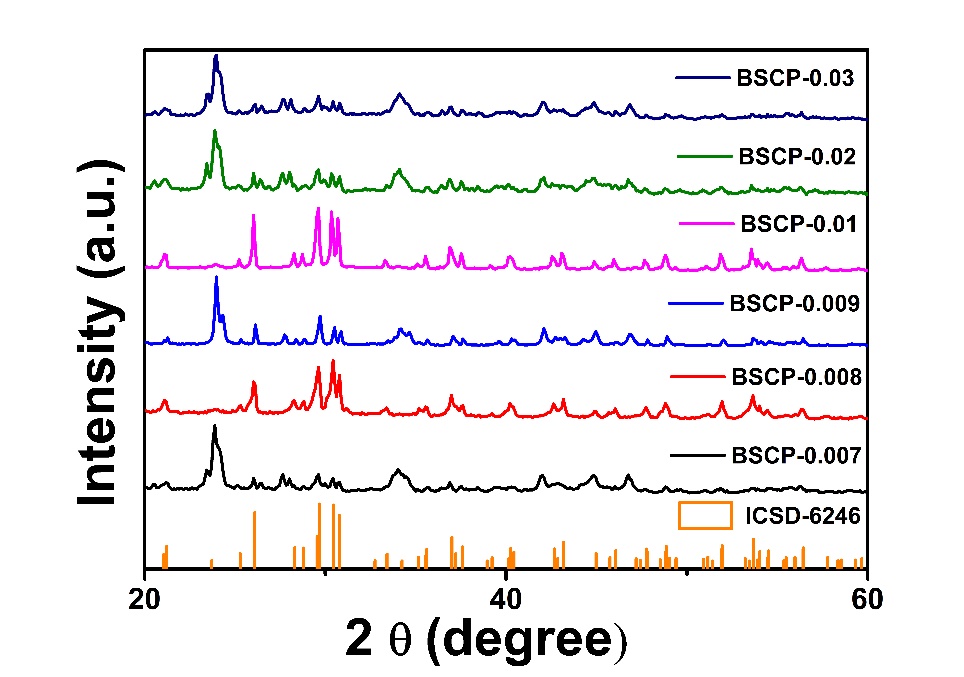


Figure S1: X-ray diffraction patterns of BSCP-0.007, 0.008, 0.009, 0.01, 0.02, and 0.03 phosphors.


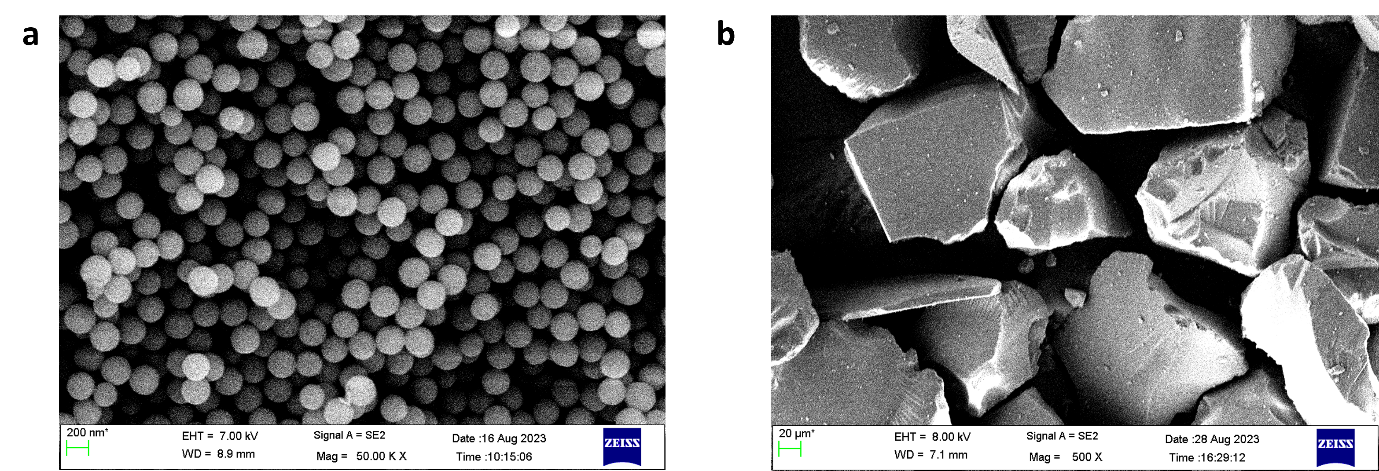


Figure S2: (a) and (b) represent FE-SEM images of silica nanoparticles and conventional silica.


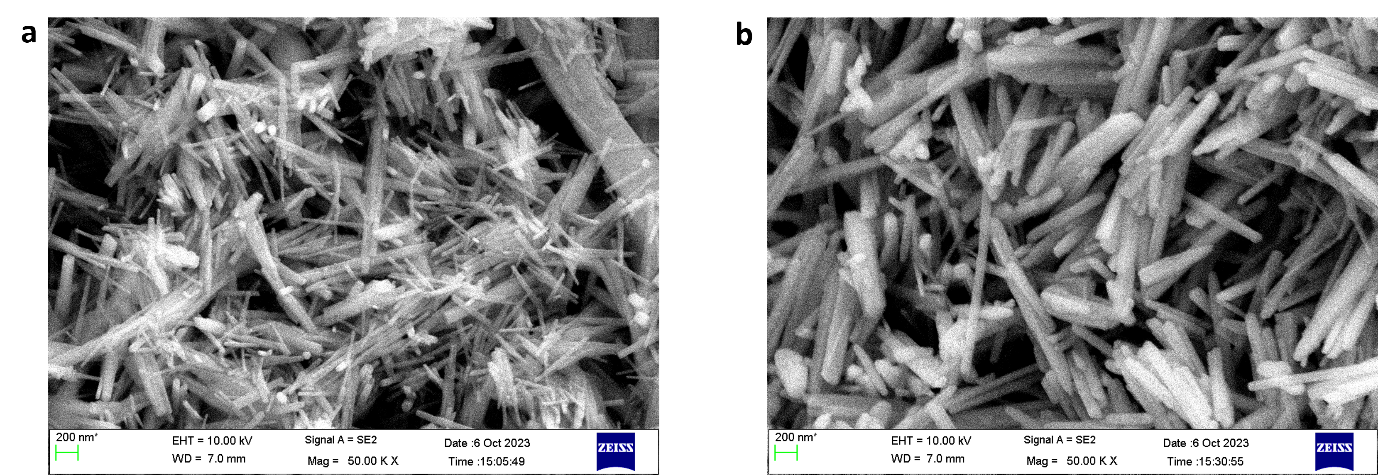


Figure S3: (a) and (b) represent FE-SEM images of BSSP-0.01 and BSCP-0.009 phosphor.


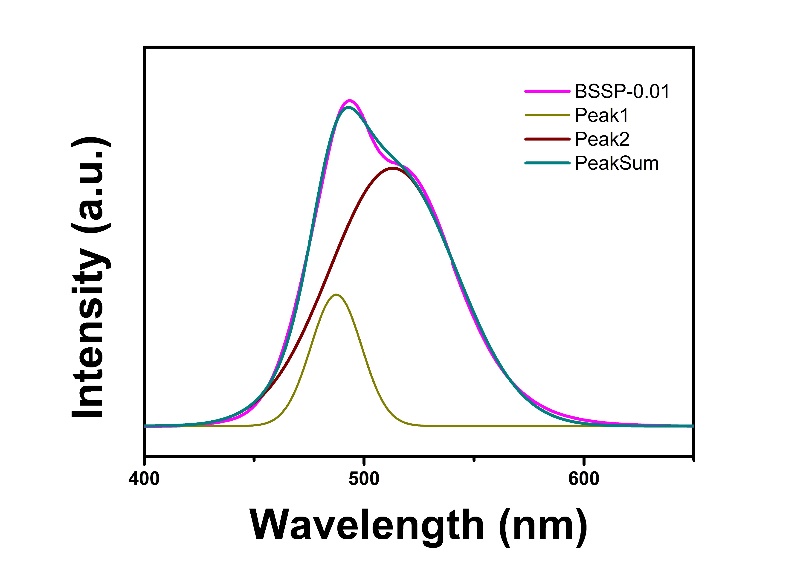


Figure S4: Gaussian fitting spectrum of BSSP-0.01 phosphor.


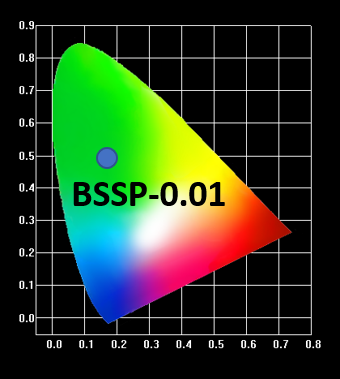


Figure S5: CIE chromaticity of optimized BSSP-0.01 phosphor.

| Weight of the phosphor  (mg) in 60 mg of binder | CIE  (x, y) |
| --- | --- |
| 30 | 0.15, 0.47 |
| 40 | 0.15, 0.47 |
| 50 | 0.15, 0.47 |
| 60 | 0.15, 0.47 |
| 70 | 0.15, 0.47 |

Table S1: Weight of the phopshor vs CIE coordinates of the BSSP remote phosphor

| **Voltage (V)** | **CIE coordinates (x, y)** |
| --- | --- |
| 2.9 | 0.25, 0.35 |
| 3.0 | 0.25, 0.35 |
| 3.1 | 0.24, 0.36 |
| 3.2 | 0.24, 0.38 |
| 3.3 | 0.23, 0.38 |
| 3.4 | 0.23, 0.39 |
| 3.5 | 0.23, 0.38 |
| 3.6 | 0.23, 0.38 |

Table S2: Applied Voltage Vs CIE coordinates of BSSP film-50 mg.

| **Change in time (h)** | **CIE coordinates (x, y)** |
| --- | --- |
| 1 | 0.23, 0.38 |
| 2 | 0.23, 0.38 |
| 3 | 0.23, 0.38 |
| 4 | 0.23, 0.38 |
| 5 | 0.23, 0.38 |
| 6 | 0.23, 0.38 |
| 7 | 0.23, 0.38 |
| 8 | 0.23, 0.38 |

Table S3: Change in time Vs CIE coordinates of optimized BSSP film – 50 mg.
